# The Late Professor Horner

**Published:** 1854-02

**Authors:** 


### THE LATE PROFESSOR HORNER.

Professor Jackson, in his introductory lecture delivered in the University of Pennsylvania, at the opening of the present session, commemorative of the late Dr. Wm. E. Horner, details some interesting and affecting incidents in the life of that eminent teacher and physician. He says:—

"It would not be doing justice to Dr. Horner, or give a correct idea of his energy, self-command, and indomitable resolution, or a true conception of the disadvantages under which he labored, the long unceasing struggles he sustained in his progress, and the heavy cost at which his success was attained, did I not reveal a peculiarity of temperament, or psychical idiosyncracy, never observed or suspected to exist, by his most intimate associates and friends, or even by a large portion of his family circle.

"While his exterior life appeared clear, bright, calm and prosperous, his interior life was dark, desponding, agitated with vague apprehensions, and every mental effort a conflict, a struggle, and a victory."

Prof. Horner, in a journal which he kept for a number of years, quoted by his biographer, thus describes his "constitution -"

"It was considered at school that I learned with facility; but I never believed it. I have had headache or dull pains in the head three-fourths of my waking life, seldom acute, but always such as to make me uncomfortable, and prefer solitude to company.

"Short intermissions of this state of suffering have occurred. I have then felt illuminated as the earth is when the sun emerges from behind a cloud. I have then hoped for a pleasurable existence, but it proved delusive, and I quickly relapsed into my ordinary state. Considering this serious obstacle to mental improvement, I wonder how I have made any advances, and especially such as to have given me an honorable station among men."

In another place he says:—

"My spirits get into so deplorable and hypochondriac a state that I have a thousand times thought death would be a most welcome visiter, and have almost envied those whom I have just heard to have passed from the bondage and anxieties of this life.

"As I grow older, my system is evidently getting more and more under the influence of the preceding causes. From the smallest article of food used in the evening, the next morning I am rendered uncomfortable in the extreme; my mental faculties are hebetated, and I am so vertiginous as scarcely to be able to collect my ideas or go on with a demonstration. The latter state has indeed become so constant and frequent, that I have frequently thought my labors as a public teacher were becoming too imperfect and confused to deserve respect, and that it would be better, perhaps, for me to retire and seek for some other occupation."

He wrote thus in his journal, in 1829:—

"I go to bed dissatisfied, taciturn, and looking for no greater comfort on the day to come, than I have enjoyed during the day past. Such is the unprepossessing picture of my life at the present time, and such has it been during the last six weeks; enjoyment has ceased, happiness has fled; I am inactive, worthless, lethargic."

And a few weeks afterwards he wrote as follows:

"I find it vain to resist the current of one's nature. I am at the present moment, just as I have been for the last four months, a confirmed and dissatisfied *ennuyé*. Discontented with myself and feeling no pleasure or satisfaction in the things around me, and finding every plan abortive, either in study, religion or amusement, from which I hoped to obtain that steady and enduring quietude of mind, which I have on former occasions enjoyed. I must now make up my mind to move down the current of life on those terms that destiny, my peculiar nature, and my particular pursuits seem to have imposed unchangeably on me. I thank my Creator for the many unmerited favors I have received, and am constantly receiving at his hands. I ask pardon and forgiveness for the ingratitude of my nature, which prevents my mind from being illuminated with a single ray of joy, in reflecting on all His goodness. In the midst of the means of happiness, I am the victim of an unhappy destiny; my mind is cast in a mould which makes it insensible to the best gifts of Providence; and all that remains for me, is to submit resignedly during the remainder of the voyage, now drawing to a close, down the overflowing stream of time."

And yet, as remarked by PROF. JACKSON, "the unhappy and disabling affliction," recorded in these memoranda, "was manifested by no exterior sign," and the fact that Dr. HORNER was the subject of such a melancholy will take by surprise those who were accustomed to see him in his daily walk. Of course, the source of his mental disquietude was some physical derangement—some morbid condition, most probably, of the brain, which was only a little less intense than that which drove the amiable COWPER to the brink of insanity. It shows the heroic character of Dr. HORNER's mind, that he was able to go forward under such a gloom in the discharge of his manifold duties without a murmur or one word of complaining.

